# Software-supported analysis of MRgFUS therapy outcome

**DOI:** 10.1186/2050-5736-3-S1-P83

**Published:** 2015-06-30

**Authors:** Doerte Corr, Florian Weiler, Martijn Hoogenboom, Martinus J van Amerongen, Jurgen Fütterer, Tetiana Dadakova, Michael Bock, Matthias Günther, Jürgen Jenne

**Affiliations:** 1 mediri GmbH, Heidelberg, Germany; 2 Fraunhofer MEVIS, Bremen, Germany; 3 Radboud University Medical Center, Nijmegen, Netherlands; 4 University Medical Center Freiburg, Freiburg, Germany

## Background/introduction

Recently, first studies on MRgFUS for therapy of localized prostate cancer have been presented. However, MRI is not only useful to monitor and guide FUS prostate cancer therapy but might add valuable morphologic and functional information for therapy stratification and outcome. Since multi-parametric MR protocols are time-consuming to generate and evaluate, they are not performed during an MRgFUS session.

Rather these data are acquired before MRgFUS for therapy planning and after therapy for outcome assessment. This approach leads to a plethora of MR images (before, during and after therapy) that can be difficult to analyze and correlate. To support the radiologist, a software prototype was therefore developed to analyze MRgFUS therapy outcome based on image registration and synchronization.

## Methods

**Figure 1:**
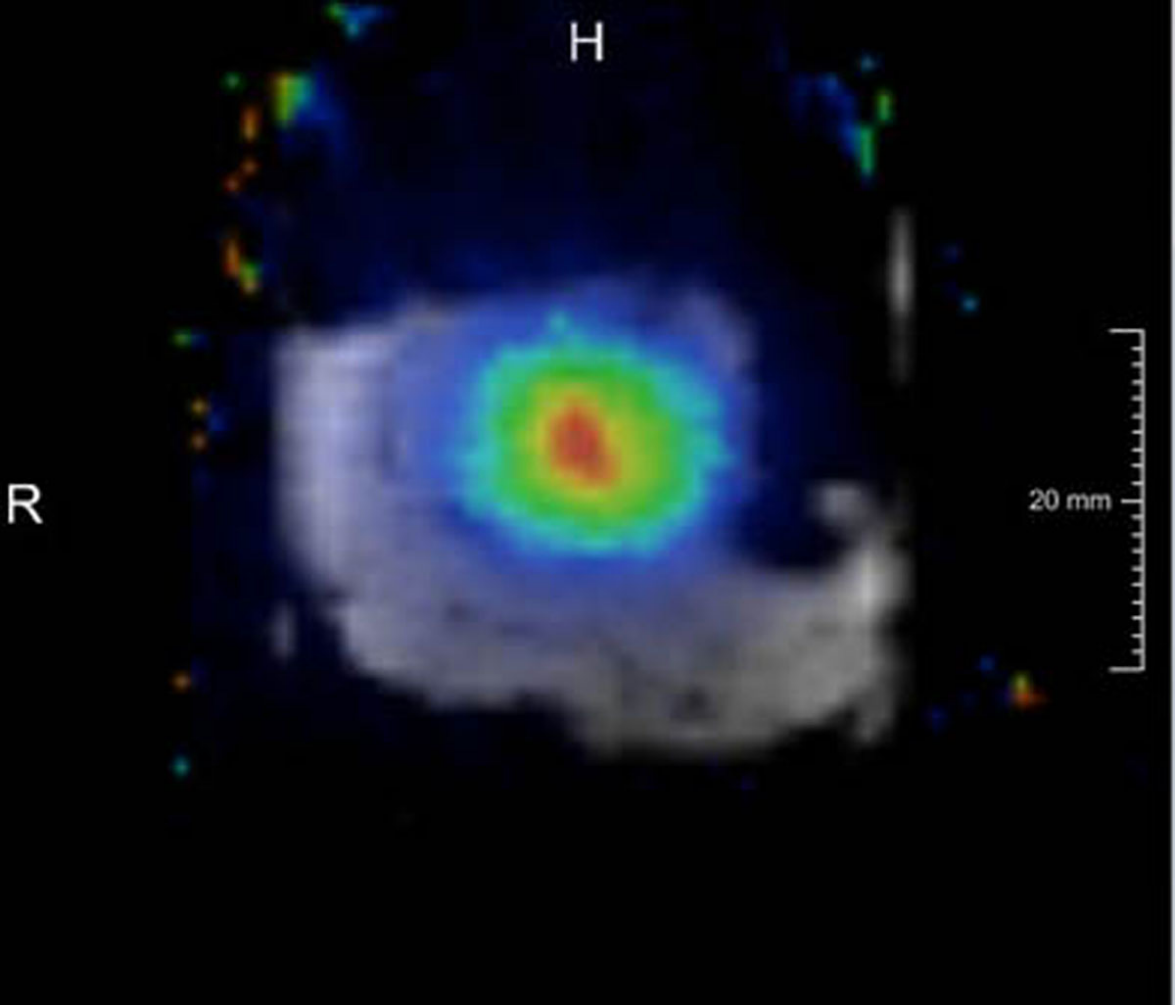
T1w MR image of a meat sample with temperature map overlay.

Initially, the workflow of MRgFUS in combination with multi-parametric MR examinations before and after therapy was analyzed. Based on the analysis, a software prototype addressing the specific workflow was built with the development environment MeVisLab. The software comprises the following functionalities: 2D viewers with basic interaction functionality (zoom, window/level modification, overlays), display of FUS temperature maps (Fig 1), image ordering and classification, automatic image registration, interactive registration refinement, synchronization cursor, contouring tool for segmentation and annotations, and ROI statistics. To analyze therapy outcome all available MR data of the patient are imported. Data are automatically ordered and classified for automatic post-processing and visualization. Next, images from different examinations are automatically co-registered. The linear registration focusses on T2w MR images which are available in every examination. If results are insufficient, registration can be manually refined. The user then selects images for correlated display which are synchronized with crosshairs.

Additionally, ROIs can be placed and tracked on follow-up images or across modalities to better discriminate between tumor recurrence and scar formation in suspicious areas. MR thermometry temperature maps can be imported as well to correlate the applied thermal dose with post-therapeutic image changes. Furthermore, contrast-enhanced images acquired at the end of a therapy session can be compared with diagnostic or follow-up images to check if all affected areas were covered.

## Results and conclusions

**Figure 2:**
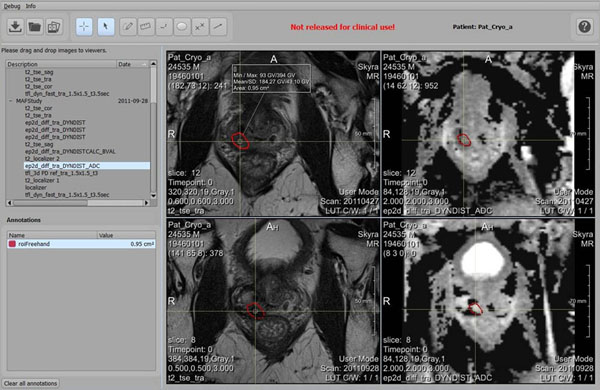
Screenshot of the software called Local Therapy Control showing pre-therapeutic (top row) and post-therapeutic (bottom row) prostate T2w and ADC images and a tracked ROI. Images are synchronized at the cross-hair position based on a registration.

A stable version of the software was released for clinical testing. First tests were performed on anonymized data of prostate cancer patients who had extensive MR imaging prior to and past cryo therapy (Fig 2), as well as on meat data treated with FUS. The software implements all necessary features for analyzing the therapy outcome and correlation with previous diagnostic and therapeutic data. In addition it could be used for therapy planning. Further evaluation of the software, including a registration accuracy evaluation, is currently in preparation.

